# Dataset on households’ perceptions, waste sorting and contributions for improved domestic waste management in Vietnam: An application of culture tower and CVM

**DOI:** 10.1016/j.dib.2026.113286

**Published:** 2026-09-24

**Authors:** Van Quy Khuc, Ngoc Duc Doan, Thi Vinh Ha Nguyen

**Affiliations:** a Faculty of Development Economics, VNU University of Economics and Business, Vietnam National University, Hanoi 100000, Vietnam; b SCDM lab, VietKAP, Hanoi 100000, Vietnam

**Keywords:** Waste management, Household perception, Waste sorting, Willingness to pay, Culture tower, CVM, Vietnam

## Abstract

Developing effective solutions for domestic waste management to achieve cleaner living environments represents a critical priority for many developing nations. This research aims to comprehensively understand Vietnamese citizens’ perceptions of household waste management challenges and their behavioral responses, with particular emphasis on their willingness to financially contribute toward enhanced waste management services. To be specific, between June and July 2025, we employed a hybrid data collection methodology integrating digital and in-person techniques, utilizing a stratified random sampling approach combined with the Contingent Valuation Method (CVM) to survey 414 households across 7 communes/wards in Hai Phong City, Vietnam. The study utilized the Culture Tower (CT) to systematically design and collect comprehensive data encompassing four primary information categories: (1) perceptions and attitudes toward waste management challenges and satisfaction levels with current waste treatment services, (2) existing household waste separation behaviors and practices, (3) willingness-to-pay commitments for improved waste management services and underlying motivational factors, (4) sociodemographic and economic characteristics of respondents. The study's findings provide valuable empirical evidence that can inform evidence-based policy development for enhanced waste management systems in the research area and beyond.

Specifications Table

**Table:** 

Subject	Social Sciences
Specific subject area	Environmental Science, Environment management
Type of data	Table Figures Excel files
How data were acquired	Data were obtained from field surveys conducted in June–July 2025 combining face-to-face interviews with an online questionnaire administered via Google Forms. The responses were compiled in Microsoft Excel (.xlsx) format for statistical analysis in Stata version 17.0 and the R statistical environment (R version 4.5.2)
Data format	Raw Analyzed
Description of data collection	The data in this study came from 414 households in 7 communes/wards in Hai Phong City, Vietnam, selected using a stratified random sampling approach combined with the Contingent Valuation Method. The questionnaire captured socio-demographic characteristics and key dimensions of household waste management, including perception factors, behavioral factors, satisfaction factors, and willingness to pay (WTP) for improving waste management services.
Data source location	Information was collected from Hai Phong City (Latitude 20° 51′ 0″ N, Longitude 106° 40′ 0″ E), Vietnam
Data accessibility	Repository name: Mendeley repository Data identification number: DOI: 10.17632/wzzjzkstyz.4 Direct URL to data: https://data.mendeley.com/datasets/wzzjzkstyz/4

## 1 Value of Data

• The dataset provides a comprehensive foundation for studying environmental behavior, particularly households’ willingness and capacity to financially contribute to domestic waste management. It enables the identification of key determinants influencing environmental actions and community engagement in waste management initiatives, thereby supporting evidence-based strategies for cleaner living environments. In addition, the data allow for an in-depth examination of environmental cultural dimensions based on the Culture Tower, covering multiple levels from perception to behavioral participation in waste sorting, thereby enabling a systematic evaluation of environmental culture development.The dataset is valuable for policy evaluation and academic research. It enables the assessment of the effectiveness of source waste separation policies implemented in Vietnam since January 2025 and can be used for cross-country comparisons or to replicate similar studies in different contexts. Moreover, it serves as a practical resource for teaching environmental statistics and applied data analysis.
• The dataset also provides a structured questionnaire and variable framework that can guide future research designs, methodological enhancements, and reproducibility in community-based waste management and environmental behavior studies.

## 2 Background

In the context of rapid economic growth, emerging economies such as Vietnam are increasingly facing serious environmental challenges. Among these, the management of municipal solid waste (MSW) has become a particularly pressing issue, with approximately 60,000 tons generated daily over 60% originating from major urban areas [1].

Hai Phong, known as Vietnam’s second-largest port city and a key industrial and logistics hub, plays a crucial role in this context, producing about 1800 tons of waste per day. As part of the Red River Delta region, the city has undergone recent administrative expansion, now covering approximately 3194.72 km² with a population of about 4.66 million (Fig. 1) [2,3]. This expansion has intensified environmental pressures, particularly on household waste collection, treatment, and recycling, increasing the complexity and cost of local waste management systems.

**Fig. 1:**
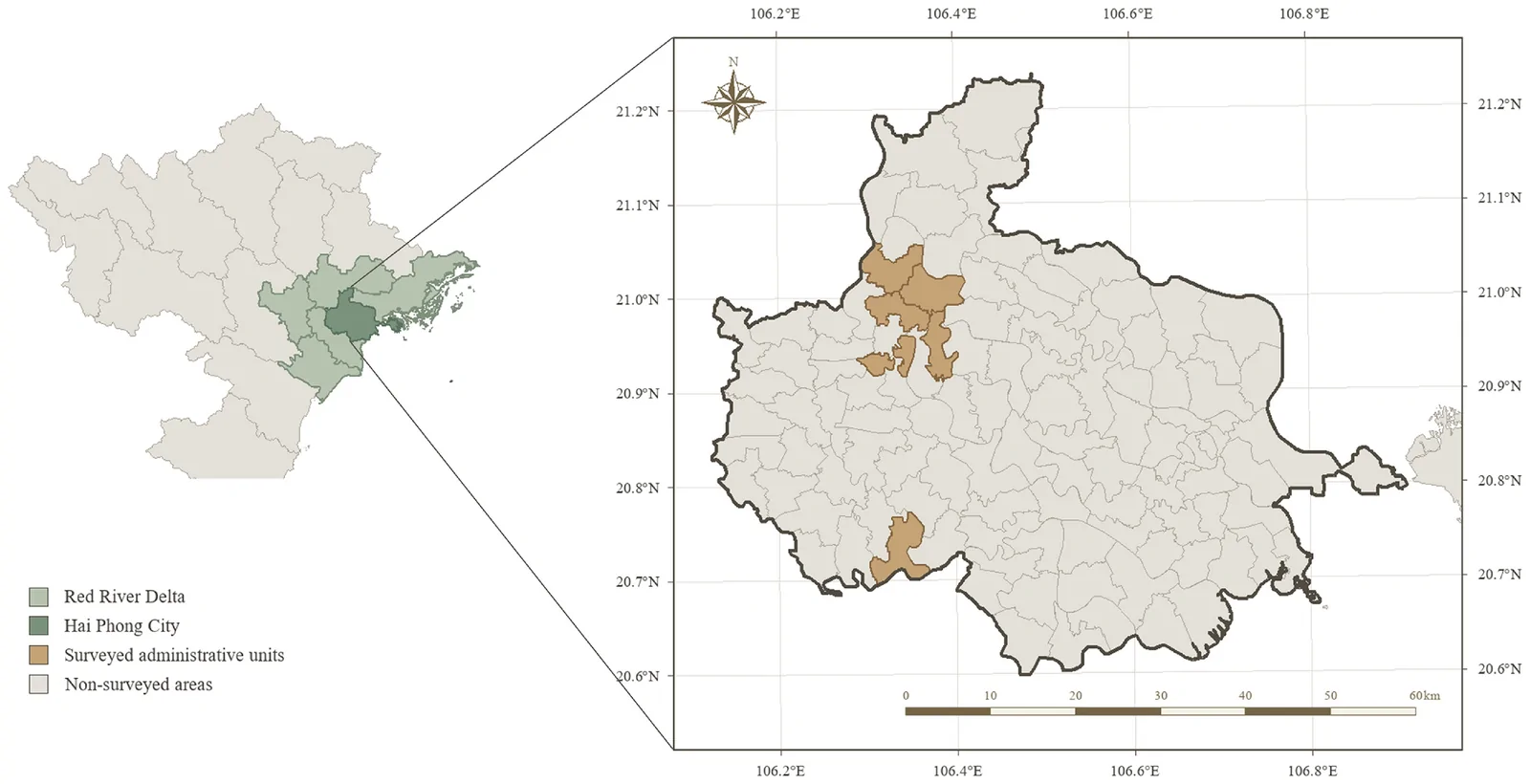
Location of the study area in Hai Phong City, Vietnam.

To respond to the escalating pressure on waste collection and treatment systems, the 2020 Law on Environmental Protection introduced Article 75, which mandates waste separation at source starting from January 1, 2025 [4]. This regulation aims to enhance collection and treatment efficiency, promote the transition toward a circular economy, and strengthen public participation in household waste management. However, implementation remains inconsistent, with reports indicating continued mixed waste disposal in many areas [5].

Previous studies in Vietnam indicate that households’ willingness to pay (WTP) and waste management behaviors vary across regions and are influenced by socio-economic characteristics [6,7]. However, empirical data on newly expanded metropolitan areas, such as Hai Phong, remain limited.

In view of these gaps, the development of an original quantitative dataset focusing on household-level awareness, waste-sorting behaviours, willingness to contribute financially and associated socio-economic factors in the expanded Hai Phong region becomes imperative. The selected communes/wards were deliberately chosen as localities that have been at the forefront of implementing waste separation at source policies, thereby providing a relevant empirical setting to examine early-stage behavioural responses to recent regulatory changes. The spatial distribution of the study sites, including one area located further south, reflects the stratified sampling design and ensures coverage of diverse socio-economic and urban–peri‑urban conditions across the expanded city. Such data provides crucial insights into behavioural patterns that can inform policy design, support local implementation of the source‐separation legislation, and contribute to comparative research across developing countries facing similar environmental management challenges.

## 3 Data Description

In this section, we present the dataset using tables and figures. There are five tables and one figure representing five main groups of information: demographic characteristics, perceptions of waste pollution, behavioral practices, satisfaction with waste management services, and perceived causes of increasing local pollution.

Table 1 below presents the demographic characteristics of the surveyed households and their corresponding mean willingness to pay (WTP). The sample includes 414 respondents, with a clear female majority (82.1%) and most participants aged 31–50 years. In terms of education, bachelor’s degree holders form the largest group (40.3%), followed by those with secondary or high-school education. Average WTP remains modest across demographic groups. Younger and better-educated respondents tend to show higher mean WTP, while no noticeable difference is observed between male and female participants.

**Table 1:** Households’ WTP for improved domestic waste management by their demographic factors.

Demographic factors				WTP ($)				
Variables	Definitions	Options	Percentages	Mean	S.D	S.E	Min	Max
Gender	Respondent’s gender	Male	17.9	1.7	2.9	0.3	0	19.2
		Female	82.1	1.8	2.5	0.1	0	19.2
Age	Age group of respondent	21 – 30	3.4	2.4	2.9	0.8	0	7.7
		31 – 40	30.9	2.1	3.4	0.3	0	19.2
		41 – 50	32.1	1.6	1.9	0.2	0	7.7
		51 – 60	20.0	1.7	2.6	0.3	0	19.2
		Over 60	13.5	1.0	1.4	0.2	0	6.2

Education	Highest level of education attained	Secondary school or below	23.4	1.4	1.9	0.2	0	7.7
		Highschool	20.3	1.5	2.6	0.3	0	19.2
		Technical school/College degree	10.4	1.0	0.9	0.1	0	3.8
		Bachelor's Degree	40.3	2.0	2.8	0.2	0	19.2
		Master's Degree	5.6	2.9	3.9	0.8	0	19.2
		Doctoral Degree	0	0	0	0	0	0

**Notes:** Reported WTP amounts represent monthly household contributions expressed in U.S. dollars, calculated using an exchange rate of 26,000 VND per USD. The dataset contains 414 respondents with valid WTP entries. All percentage values are expressed in percent (%), and are calculated based on valid responses for each variable. S.D. and S.E. denote the standard deviation and standard error, respectively.

Table 2 presents households’ perceptions of local waste pollution and their corresponding mean willingness to pay (WTP). Most households report no unpleasant odor from nearby collection sites (81.2%), and more than half perceive that illegal dumping occurs rarely (53.6%). Around two-thirds feel only moderately or normally affected by waste-related pollution, and most consider the current collection fee reasonable. Across perception groups, WTP show limited variation overall, but certain differences are evident. Respondents who perceive odors, frequent dumping, or worsening pollution express a relatively stronger willingness to pay for service improvement, while those who feel unaffected or satisfied with current fees exhibit less readiness to contribute.

**Table 2:** Households’ WTP for improved domestic waste management by their perceptions.

Perception factors				WTP ($)				
Variables	Definitions	Options	Percentages	Mean	S.D	S.E	Min	Max
OdorDetect	Household perception of odor presence from nearby waste collection sites	No	81.2	1.5	2.4	0.1	0	19.2
		Yes	18.8	2.6	3.1	0.4	0	19.2
OdorFrequency	Frequency of odor occurrence detected by the household	Not often	74.2	1.8	2.6	0.2	0	19.2
		Normal	21.4	1.4	2.7	0.3	0	19.2
		Regular	4.4	3.0	5.2	1.4	0	19.2
IllegalDumpingLevel	Evaluation of the level of illegal household waste dumping in public or vacant areas	Occurs frequently	28.5	2.3	3.0	0.3	0	19.2
		Occurs rarely	53.6	1.6	2.5	0.2	0	19.2
		Does not occur	17.9	1.1	1.7	0.2	0	7.7
PollutionImpact	Degree to which environmental pollution from household waste affects daily life	Strongly affected	23.4	2.0	2.9	0.3	0	19.2
		Affected	37.8	2.0	3.0	0.3	0	19.2
		Normal	30.1	1.6	2.6	0.3	0	19.2
		Not affected	8.6	0.7	0.8	0.2	0	3.8
PollutionVsPast	Perceived change in the level of household waste pollution compared to previous years	Getting worse	8.5	2.7	4.1	0.8	0	19.2
		No change	24.2	1.1	1.5	0.2	0	7.7
		Getting better	67.3	1.9	2.8	0.2	0	19.2
FeeReasonable	Perceived reasonableness of the current monthly waste collection fee	Very unreasonable	3.9	1.1	1.8	0.5	0	7.7
		Unreasonable	2.4	1.0	1.6	0.5	0	4.6
		Normal	42.5	1.9	2.4	0.2	0	19.2
		Reasonable	47.8	1.6	2.8	0.2	0	19.2
		Very reasonable	3.4	1.1	1.5	0.4	0	3.8

**Notes:** Reported WTP amounts represent monthly household contributions expressed in U.S. dollars, calculated using an exchange rate of 26,000 VND per USD. The dataset contains 414 respondents with valid WTP entries. All percentage values are expressed in percent (%), and are calculated based on valid responses for each variable. S.D. and S.E. denote the standard deviation and standard error, respectively.

Table 3 presents households' behavioral practices related to waste management and their corresponding mean willingness to pay (WTP). Most respondents indicate that waste collection generally follows the designated schedule (81.6%) and that transportation activities comply with regulations (87.2%). Over half of households sort waste at source (55.1%), while public waste bins are available in approximately one-third of residential areas. In general, average WTP does not vary greatly across behavioral groups, but households engaging in proper waste-sorting practices or living in areas with public bins tend to value service improvement more highly. This pattern suggests that positive waste-management behavior coincides with greater willingness to contribute financially.

**Table 3:** Households’ WTP for improved domestic waste management by their behavioral factors.

Behavioral factors				WTP ($)				
Variables	Definitions	Options	Percentages	Mean	S.D	S.E	Min	Max
ScheduleAdherence	Compliance of waste collection personnel with the designated collection schedule	No	11.4	2.1	1.9	0.3	0	7.7
		Yes	81.6	1.6	2.5	0.1	0	19.2
		Don’t remember	7.0	2.0	3.9	0.7	0	19.2
SourceSort	Whether waste is sorted at source before being transported to the transfer station	No	26.1	1.0	1.2	0.1	0	7.7
		Yes	55.1	2.0	2.7	0.2	0	19.2
		No opinion	18.8	1.9	3.3	0.4	0	19.2
TransportCompliance	Compliance of waste transportation with designated aggregation and transfer regulations	No	3.6	0.4	0.6	0.2	0	1.5
		Yes	87.2	1.7	2.3	0.1	0	19.2
		Don’t know	9.2	2.4	4.5	0.7	0	19.2
PublicWasteBins	Existence of public waste bins in the residential area	No	62.6	1.5	2.5	0.2	0	19.2
		Yes	33.1	2.1	2.6	0.2	0	19.2
		Don’t know	4.3	1.5	2.3	0.5	0	7.7

**Notes:** Reported WTP amounts represent monthly household contributions expressed in U.S. dollars, calculated using an exchange rate of 26,000 VND per USD. The dataset contains 414 respondents with valid WTP entries. All percentage values are expressed in percent (%), and are calculated based on valid responses for each variable. S.D. and S.E. denote the standard deviation and standard error, respectively.

Table 4 presents households’ satisfaction with local waste collection and treatment services and their corresponding mean willingness to pay (WTP). Most respondents' express neutral to positive satisfaction with both collection and treatment activities. Only a small share of respondents reports being very dissatisfied or very satisfied. In terms of WTP, households that are less satisfied with current services tend to indicate higher payment intentions, while those who are content with existing conditions show lower WTP. This pattern reflects that perceived service shortcomings may encourage greater willingness to support improvements

**Table 4:** Households’ WTP for improved domestic waste management by satisfaction factors.

Satisfaction factors				WTP ($)				
Variables	Definitions	Options	Percentages	Mean	S.D	S.E	Min	Max
SatisfactionCollection	Level of satisfaction with local waste collection activities	Very dissatisfied	2.9	0.6	0.5	0.1	0	1.5
		Disssatisfied	8.0	3.7	3.6	0.6	0	19.2
		Normal	48.3	1.6	2.2	0.2	0	19.2
		Satisfied	35.7	1.4	2.2	0.2	0	19.2
		Very satisfied	5.1	1.9	4.4	1.0	0	19.2

SatisfactionTreatment	Level of satisfaction with local waste treatment activities	Very dissatisfied	1.9	3.1	6.5	2.3	0	19.2
		Disssatisfied	8.9	3.5	3.5	0.6	0	19.2
		Normal	55.1	1.6	2.3	0.2	0	19.2
		Satisfied	29.7	1.4	2.2	0.2	0	19.2
		Very satisfied	4.3	0.7	1.3	0.3	0	3.8

**Notes:** Reported WTP amounts represent monthly household contributions expressed in U.S. dollars, calculated using an exchange rate of 26,000 VND per USD. The dataset contains 414 respondents with valid WTP entries. All percentage values are expressed in percent (%), and are calculated based on valid responses for each variable. S.D. and S.E. denote the standard deviation and standard error, respectively.

Table 5 below illustrates households’ perceptions of the major causes behind rising local waste pollution. Respondents mainly attribute the problem to human behavior and lifestyle changes, particularly limited public awareness and increasing daily consumption. Structural issues such as inadequate infrastructure or industrial waste are mentioned less frequently. Overall, the findings highlight a prevailing view that improving public awareness and everyday waste practices is more urgent than solely expanding physical waste-management capacity.

**Table 5:** Perceived causes of increasing local waste pollution.

Dimensions	N	Percentages
Population growth	172	41.5%
Increasing living demands	218	52.7%
The development of production and daily activities increases waste generation	242	58.5%
Limited public awareness	308	74.4%
Industrial waste increases pollution	174	42.0%
Household waste increases pollution	218	52.7%
Low infrastructure	169	40.8%
Other	0	0

**Note:** Interviewees can choose multiple causes.

Fig. 2 below summarizes key characteristics of the surveyed households, including income distribution, waste-sorting behavior, perceptions of pollution impacts, and perceived quality of local waste management services, alongside descriptive patterns of willingness to pay (WTP). The income distribution indicates that most respondents are concentrated in the middle-income ranges, particularly between 8 and 30 million VND per month, where the number of observations is highest. Mean WTP generally increases with income and reaches its highest level among households earning 40–50 million VND per month, before declining at higher income levels where sample sizes are small. With respect to environmental practices, slightly more than half of respondents' report sorting household waste at source, while the remainder do not engage in this practice. Perceptions of environmental conditions show that the majority of respondents consider themselves either affected or strongly affected by pollution, whereas a smaller proportion report being unaffected. In terms of service evaluation, perceived quality of local waste management services is mainly concentrated in the mid-to-upper range of the scale (approximately 5–8 points), with fewer observations at the lower and upper extremes. Overall, the figure provides a descriptive overview of how income levels, household environmental practices, pollution perceptions, and service quality assessments are distributed within the sample, offering contextual background for understanding variations in respondents’ stated willingness to pay for improvements in waste management services.

**Fig. 2:**
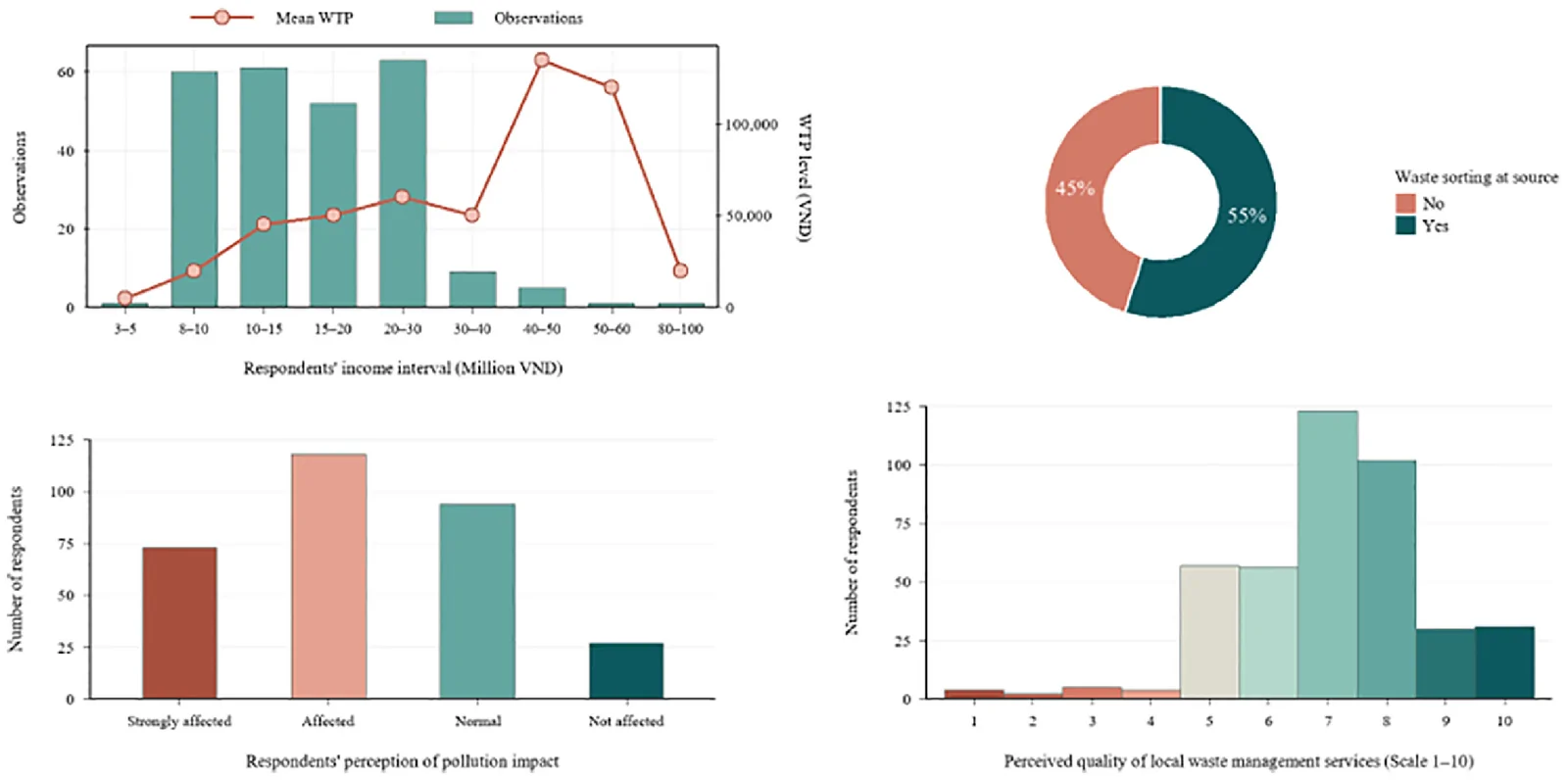
Willingness to pay (WTP) for improved waste management services by household behaviors and perceptions.

## 4 Experimental Design, Materials and Methods

### 4.1 Survey design and data collection

The dataset was developed through a structured household survey conducted in Hai Phong, Vietnam, between June and July 2025. The survey aimed to collect quantitative information on households’ perception, satisfaction, behavioral practices, and willingness to pay (WTP) for improved domestic waste management services. Hai Phong was selected as the study area due to its rapid urbanization, high waste generation, and early adoption of mandatory waste separation at source starting in 2025. An integrated data collection approach was adopted, combining face-to-face interviews and online surveys to ensure inclusiveness and data reliability. The study employed stratified random sampling, with strata defined by residential location (urban and rural), to ensure representativeness across socio-economic and geographic groups. In total, 414 valid household responses were collected from seven communes/wards within the city. All contacted households agreed to participate in the survey, resulting in a response rate of 100% for the realized sample with no recorded refusals.

The questionnaire was designed based on the Culture Tower [8,9]. To ensure contextual appropriateness, the questionnaire was pilot-tested and subsequently refined during both the pilot phase and the main survey implementation. This questionnaire focused on four main information groups: (i) perceptions and attitudes toward waste management challenges and satisfaction with current services; (ii) household behaviors and practices in waste separation at source; (iii) willingness-topay commitments and motivational factors for service improvement; and (iv) sociodemographic and economic characteristics of the respondents. The findings from this survey provide valuable empirical evidence to inform data-driven policy development for waste management in Hai Phong and other major cities in Vietnam.

### 4.2 Data processing and coding

All survey responses were checked, cleaned, and coded prior to analysis. The dataset was first reviewed for completeness and consistency; incomplete or invalid responses were excluded, resulting in a total of 414 valid observations. A total of 437 respondents were initially approached, yielding an overall response rate of 94.7%. Data entry was carried out using a standardized coding template based on the questionnaire design and verified through double‐entry checks to minimize transcription errors.

Categorical variables were converted into numerical codes following the predefined scales in the codebook. The measurement scales used in the questionnaire were developed by combining items adapted from existing literature on environmental perception and service evaluation with context-specific questions tailored to the local waste management conditions in Hai Phong [10]. Specifically, binary variables were coded as 0 and 1, while ordinal and Likert‐type variables used 3–5‐point or 10‐point numeric scales according to question format. The five major groups of variables include: (A) demographic characteristics; (B) perception of household waste pollution; (C) waste collection and treatment practices; (D) satisfaction with local waste services; (E) willingness to pay (WTP) for improved services.

For the WTP variables, WTP was coded as a binary variable (1 = “Yes, willing to pay”; 0 = “No”), while WTPamount represented the maximum monthly contribution selected by respondents using the payment‐card method. Monetary values were originally recorded in Vietnamese dong (VND) and converted to U.S. dollars using an exchange rate of 26,000 VND = 1 USD. A review of item-level responses indicates that most variables achieved complete response rates (100%). Limited missing values were observed in a small subset of perception-related variables, primarily due to item-specific nonresponse or non-applicable conditions. All missing values were consistently coded as “NA” in the dataset. No imputation was performed in order to preserve the integrity of the original responses. The final dataset is stored in XLSX format, containing 414 rows (households) and 22 variables with descriptive labels and value codes documented in the accompanying codebook.

### 4.3 Application

The dataset can be used for many models related to the study’s objectives. In this section/paper, we take CVM as an example of the dataset’s application. Specifically, the dataset serves as input for the CVM using the interval regression model to estimate households' willingness to pay (WTP) for improving waste management services. It contains variables describing household perceptions, waste management practices, satisfaction with existing services, and sociodemographic characteristics, providing an empirical basis for WTP modeling. Because WTP was elicited using a payment-card approach, respondents reported a range of acceptable payment values rather than a single amount, resulting in interval-censored data. For such data, conventional linear regression models are inappropriate, as they do not account for interval censoring and may yield biased estimates [11]. An interval regression model is therefore suitable, as it treats the latent WTP as a continuous variable constrained within the reported lower and upper bounds. Under a log-normal specification, expected WTP values can be obtained through back-transformation of the estimated parameters.

The interval regression model treats the unobserved (latent) true WTP as a continuous variable that lies within an interval specified by each respondent. Its log-linear specification can be expressed as:

$$\text{log}\left({\text{WTP}}_{i}^{T}\right)=\beta^{\prime }x_{i}+\varepsilon_{i}$$

Where log (${\text{WTP}}_{i}^{T}$) is the natural logarithm of the true (latent) WTP, $x_{i}$ is a vector of explanatory variables, β is a vector of parameters to be estimated, ε_i_ ∼ N (0, σ^2^) is a normally distributed error term. The expected value of WTP is then calculated using the formula:

$$E\left(\text{WTP}\right)=\text{exp}\left({\hat{\beta }}^{\prime }x_{i}\right)\cdot \exp\left(\frac{{\hat{\sigma }}^{2}}{2}\right)$$

This specification allows the model to estimate mean WTP while accommodating interval-censored data and non-negative monetary responses.

For empirical estimation, the model can be implemented in R using the “survival” package. The dependent variable is defined as an interval between the lower (Low) and upper (High) bounds of reported WTP. The estimation follows a lognormal specification, with explanatory variables representing demographic, perceptual, and behavioral factors, as shown below:

$$\begin{array}{ccc} & & \text{WT}P_{i}=\beta_{0}+\beta_{1}\text{Ag}e_{i}+\beta_{2}\text{Gende}r_{i}+\beta_{3}\text{Educatio}n_{i}+\beta_{4}\text{Incom}e_{i}+\beta_{5}\text{SourceSor}t_{i}+\beta_{6}\text{OdorDetec}t_{i}\\ & & +\beta_{7}\text{WasteServiceRatin}g_{i}+\varepsilon_{i} \end{array}$$

The following R commands provide a reproducible implementation of the model (Box 1):

Box 1 **R commands for interval regression estimation**

> library(readr)

> library(survival)

> d <- read.csv("D:/Data in brief/DIB_paper.csv")

> y <- Surv(time = d$Low, time2 = d$High, type = "interval2")

> fit_log <- survreg(y ∼ Age + Gender + Education + Income + SourceSort + OdorDetect + WasteServiceRating, data = d, dist = "lognormal")

> mu_hat <- as.numeric(predict(fit_log, type = "lp"))

> sigma <- fit_log$scale

> wtp_mean_i <- exp(mu_hat + 0.5 * sigma^2)

> wtp_mean_overall <- mean(wtp_mean_i, na.rm = TRUE)

> summary(fit_log)

Alt-text: Unlabelled box dummy alt text

The interval regression results are summarized in Table 6. The estimation shows that income, education, waste-sorting behavior, and perception of odor from nearby waste sites have positive and statistically significant effects on households’ willingness to pay (WTP) for improved waste management services. In contrast, age and waste service rating are negatively associated with WTP, suggesting lower payment intentions among older respondents and those more satisfied with current services. Gender does not appear to have a significant impact. Overall, the model demonstrates good explanatory performance (log-likelihood = –434.2), with an estimated mean WTP of approximately 58,300 VND (∼2.22 USD) per household per month**.**

**Table 6:** Estimated results of interval regression model of households’ willingness to pay (WTP) for improved waste management services.

Variables	Coefficient	Std. Error	z-value	**p-value**
Age	−0.1530	0.0569	−2.69	0.0072 ***
Gender	0.1826	0.1496	1.22	0.2222
Education	0.0847	0.0464	1.83	0.0677 *
Income	0.1859	0.0404	4.61	<0.001 ***
SourceSort	0.2741	0.1172	2.34	0.0194 **
OdorDetect	0.2636	0.1394	1.89	0.0585 *
WasteServiceRating	−0.0600	0.0354	−1.70	0.0898 *
Constant	9.5624	0.4963	19.27	<0.001 ***
Sigma = 0.847 Log-likelihood = −434.2		#Observation = 414 Mean WTP = 58,303.61		

**Note:** Dependent variable represents household WTP interval (Low, High). The model assumes a lognormal distribution of WTP. *, **, *** denote significance at 10%, 5%, and 1% levels, respectively. The unit of mean WTP value is VND.

Fig. 3 below illustrates the predicted mean WTP values by key behavioral and perceptual factors. Households that practice waste sorting display a clearly higher WTP than those that do not, indicating that environmentally responsible behavior aligns with greater readiness to contribute financially. Similarly, respondents who perceive odors from nearby waste sites exhibit notably higher mean WTP levels, reflecting that direct exposure to pollution enhances concern and willingness to support service improvement. These visual patterns are consistent with the regression findings, confirming the significant behavioral and perceptual drivers of payment intention.

**Fig. 3:**
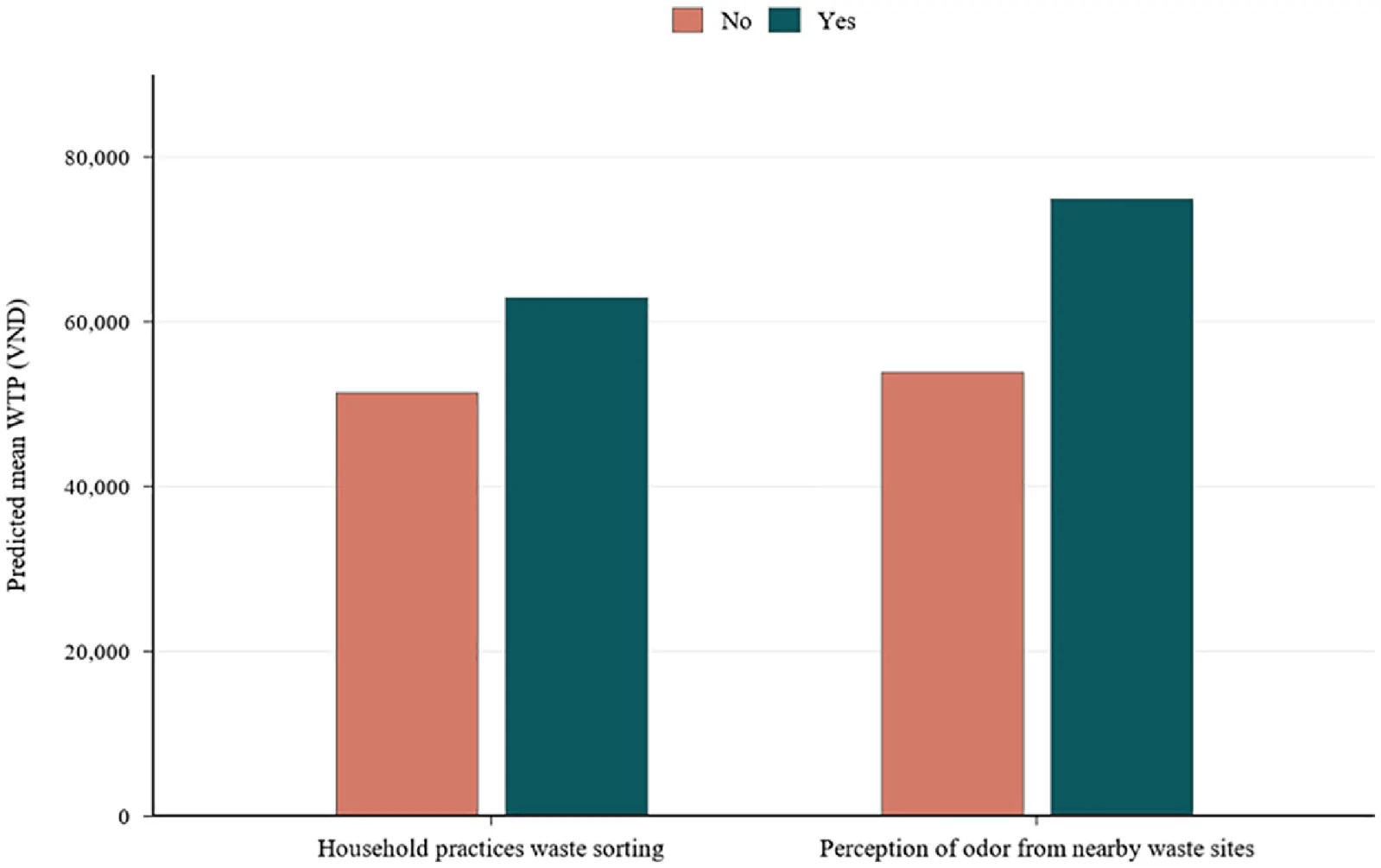
Predicted mean willingness to pay (WTP) by household waste-sorting behavior and perception of odor from nearby waste sites.

### Limitations

It is important that some limitations of our paper and/or data be noted. First, the data were collected through a contingent valuation survey, which may be subject to hypothetical bias and social desirability bias, potentially influencing respondents’ stated WTP. As a result, reported WTP values may be overestimated compared to actual payment behavior, and should therefore be interpreted with caution when used for policy simulation or economic valuation. Second, the sample is limited to a specific geographic area, which may restrict the generalizability of the findings to other regions with different socioeconomic or environmental conditions. Accordingly, caution is required when applying the dataset to cross-regional comparisons or broader generalizations beyond the study context. Lastly, as the dataset represents a cross-sectional survey, it cannot capture temporal dynamics or long-term behavioral changes in waste management practices. This limits the ability to assess causal relationships or changes over time, and users should consider this when conducting longitudinal or predictive analyses.

## Ethics Statement

Respondents to questionnaire participated voluntarily. Also, all personal information such as name, identity is not collected to ensure the respondent's privacy.

## CRediT Author Statement

**Van Quy Khuc:** Conceptualization, Methodology, Data curation, Visualization, Writing- Original draft preparation, Writing- Reviewing and Editing. **Ngoc Duc Doan:** Methodology, Data curation, Visualization, Writing- Original draft preparation, Writing- Reviewing and Editing. **Thi Vinh Ha Nguyen:** Writing- Original draft preparation, Writing- Reviewing and Editing.

## Declaration of Competing Interest

The authors state that they have no financial interest or a competitive personal relationship that has in this article.

## Data Availability

Initial dataset on households’ perceptions, waste sorting and contributions for improving domestic waste management in Red River Delta region, Vietnam (Original data) (Mendeley Data) (DOI:10.17632/wzzjzkstyz.4)
